# Artificial Intelligence Applications in Obstetric Risk Prediction: A Systematic Review of Machine Learning Models for Preeclampsia

**DOI:** 10.7759/cureus.83961

**Published:** 2025-05-12

**Authors:** Nagla Osman Mohamed Dkeen, Madina Eltayeb Dawelbait Radwan, Israa Ali Alnaw Zumam, Nihal Ahmed Abd Elfrag Mohamed, Eman Mohammed Abbashar Abdelmahmoud, Nisrin Magboul Elfadel Magboul

**Affiliations:** 1 Obstetrics and Gynecology, Najran Armed Forces Hospital, Ministry of Defense Health Services, Najran, SAU; 2 Obstetrics and Gynecology, Aljabal Primary Health Care Center, Jazan, SAU; 3 Obstetrics and Gynecology, Al Hammadi Hospital-Nuzha, Riyadh, SAU; 4 Obstetrics and Gynecology, Armed Forces Hospital, Ministry of Defense Health Services, Wadi Al Dawasir, SAU; 5 Obstetrics and Gynecology, Sultan Qaboos Hospital, Salalah, OMN

**Keywords:** artificial intelligence, machine learning, preeclampsia, risk prediction, systematic review

## Abstract

Preeclampsia remains a leading cause of maternal and perinatal morbidity and mortality worldwide. While traditional prediction models have shown limited accuracy, machine learning (ML) approaches offer promising alternatives by handling complex, non-linear relationships in multidimensional datasets. This systematic review evaluates the performance, methodological quality, and clinical applicability of ML models for preeclampsia prediction. Following Preferred Reporting Items for Systematic Reviews and Meta-Analyses (PRISMA) 2020 guidelines, we searched five databases (PubMed, Embase, Scopus, Web of Science, and Cochrane Library) for studies published until April 15, 2025, and included studies that developed or validated ML models predicting preeclampsia. Risk of bias was assessed using the Prediction Model Risk-of-Bias Assessment Tool (PROBAST). Eleven studies (n = 11, comprising 116,253 pregnancies) were included. Ensemble methods (XGBoost, Random Forest) demonstrated superior performance, with area under the curve (AUCs) ranging from 0.84 to 0.973. Key predictors included mean arterial pressure, prior preeclampsia, and the biomarkers placental growth factor (PlGF) and pregnancy-associated plasma protein A (PAPP-A). Seven studies (63.6%) showed low overall risk of bias, while three (27.3%) had high risk due to analytical limitations. Only three studies (27.3%) conducted external validation. ML models, particularly ensemble methods, show excellent discriminative ability for preeclampsia prediction. However, heterogeneity in predictors and limited external validation constrain clinical translation. Future research should prioritize prospective validation studies with standardized outcome definitions and predictor sets.

## Introduction and background

Preeclampsia is a multifactorial hypertensive disorder of pregnancy that typically emerges after 20 weeks of gestation and remains a major contributor to maternal and fetal morbidity and mortality worldwide [1]. Despite advances in maternal care, the early prediction of preeclampsia remains a formidable clinical challenge, largely due to its complex pathophysiology, heterogeneous presentation, and the lack of reliable first-trimester biomarkers. Clinically, it is characterized by elevated blood pressure and signs of organ dysfunction such as proteinuria, visual disturbances, headache, epigastric pain, and in severe cases, hepatic or renal impairment, thrombocytopenia, or pulmonary edema [2]. The condition is estimated to affect 2-8% of pregnancies globally, with a higher burden in low- and middle-income countries, where access to timely diagnosis and intervention may be limited [2]. Given its unpredictable onset and potentially fatal consequences, identifying at-risk women early in pregnancy is essential for implementing preventative strategies and improving outcomes [3].

Traditional risk assessment models based on maternal history and clinical features have demonstrated limited accuracy in predicting preeclampsia. In recent years, however, the integration of artificial intelligence (AI), a branch of computer science focused on building systems that can perform tasks typically requiring human intelligence, particularly machine learning (ML), a subset of AI that enables algorithms to learn from data and improve over time without being explicitly programmed into obstetric research, has opened new avenues for enhancing predictive accuracy by leveraging high-dimensional and multimodal data [4]. ML algorithms offer the capacity to automatically learn from complex datasets and detect latent patterns, surpassing conventional statistical methods in both flexibility and performance [5]. These algorithms can be supervised (trained on labeled data), unsupervised (discovering patterns in unlabeled data), or use hybrid methods, depending on the prediction task and available inputs. They can incorporate diverse inputs such as electronic health records, laboratory parameters, imaging data, and biochemical markers, allowing for dynamic and individualized risk stratification [6].

Recent studies have applied various ML techniques, ranging from random forests and gradient boosting to support vector machines and deep neural networks, to develop predictive models for early- and late-onset preeclampsia [6, 7]. Random forests and gradient boosting are ensemble learning methods that combine multiple decision trees for improved accuracy, while support vector machines and neural networks are capable of modeling complex, non-linear relationships in the data. While some models have reported promising performance with area under the curve (AUC) values exceeding 0.90, inconsistencies in study design, population characteristics, input features, and validation approaches have limited the generalizability and clinical integration of these tools [8].

This systematic review aims to synthesize and critically evaluate the current landscape of ML-based models for preeclampsia prediction. We assess the diversity of algorithms, the types of predictors employed, model performance metrics, and the methodological quality of included studies. Our goal is to identify high-performing models with clinical promise and highlight gaps that must be addressed before widespread adoption. By consolidating current evidence, this review seeks to inform the development of robust, generalizable, and clinically applicable ML frameworks for preeclampsia risk prediction in contemporary obstetric practice.

## Review

Methodology

Study Design and Aim

This systematic review was designed and conducted in accordance with the Preferred Reporting Items for Systematic Reviews and Meta-Analyses (PRISMA) 2020 guidelines [9]. The aim was to comprehensively evaluate studies utilizing ML models for the prediction of preeclampsia during pregnancy.

Eligibility Criteria

To ensure the inclusion of relevant and high-quality evidence, predefined eligibility criteria were applied. Studies were included if they investigated the application of machine learning or artificial intelligence algorithms for predicting preeclampsia in pregnant women. Eligible studies had to report on original research, including retrospective or prospective cohort studies, case-control, or cross-sectional designs. Only studies published in English and in peer-reviewed journals were considered. Studies that did not use machine learning techniques, review articles, editorials, letters, conference abstracts, animal studies, and those unrelated to the topic were excluded from the review.

Information Sources and Search Strategy

A comprehensive literature search was conducted across six major electronic databases: PubMed, Embase, Scopus, Web of Science, and the Cochrane Library. The search covered all relevant publications up to April 15, 2025. The search strategy was developed with input from a medical librarian and included both Medical Subject Headings (MeSH) and free-text keywords. Key terms included combinations of “preeclampsia,” “pregnancy-induced hypertension,” “machine learning,” “artificial intelligence,” “deep learning,” “support vector machine,” “random forest,” and “neural networks.” Boolean operators (AND/OR) were used to optimize search sensitivity and specificity. In addition to database searches, reference lists of relevant articles were manually screened to identify any additional eligible studies.

Study Selection Process

All identified records were imported into EndNote reference management software, where duplicates were automatically and manually removed. The study selection process was conducted in two phases. First, two independent reviewers screened the titles and abstracts of all retrieved articles to identify potentially relevant studies. Articles that passed this initial screening underwent full-text review. In cases where eligibility was unclear, consensus was reached through discussion or consultation with a third senior reviewer. The entire selection process was documented using the PRISMA flow diagram, including the number of articles included and excluded at each stage, along with reasons for exclusion.

Data Extraction Process

A standardized data extraction form was created and piloted by the review team. Two reviewers independently extracted data from each included study, ensuring accuracy and completeness. Extracted data included study characteristics such as author names, year of publication, country of study, and study design. Details about the machine learning model(s) used were collected, including algorithm type, sample size, data source, features used for prediction, and the timing of preeclampsia prediction. Outcomes and model performance metrics, such as area AUC, sensitivity, specificity, accuracy, precision, recall, and F1 score, were also recorded. Discrepancies in data extraction were resolved by discussion and, when necessary, adjudicated by a third reviewer.

Quality Assessment

The risk-of-bias assessment was performed using the Prediction Model Risk-of-Bias Assessment Tool (PROBAST) [10] to systematically evaluate four critical domains: participants (selection bias), predictors (measurement and relevance), outcome (definition and ascertainment), and analysis (statistical methods and handling of missing data). Two independent reviewers conducted the assessments, with any disagreements resolved through consensus or adjudication by a third reviewer. Each study was classified as having low, moderate, or high overall risk of bias based on its performance across these domains. This rigorous approach ensured methodological transparency and informed the interpretation of the predictive models' reliability.

Data Synthesis and Analysis

Due to significant heterogeneity among studies in terms of ML algorithms, input features, data sources, and reported outcome measures, a meta-analysis was not feasible. Instead, a narrative synthesis was conducted. The results from each study were summarized and compared qualitatively, with a focus on identifying high-performing models and commonly used predictors. Performance metrics such as AUC, sensitivity, specificity, and accuracy were reviewed in detail to assess the clinical potential of the various ML models. Additionally, the review considered differences in study design, model validation strategies, and limitations reported by the authors to provide context for the findings and guide future research directions.

Results

Study Selection Process

**Figure 1:**
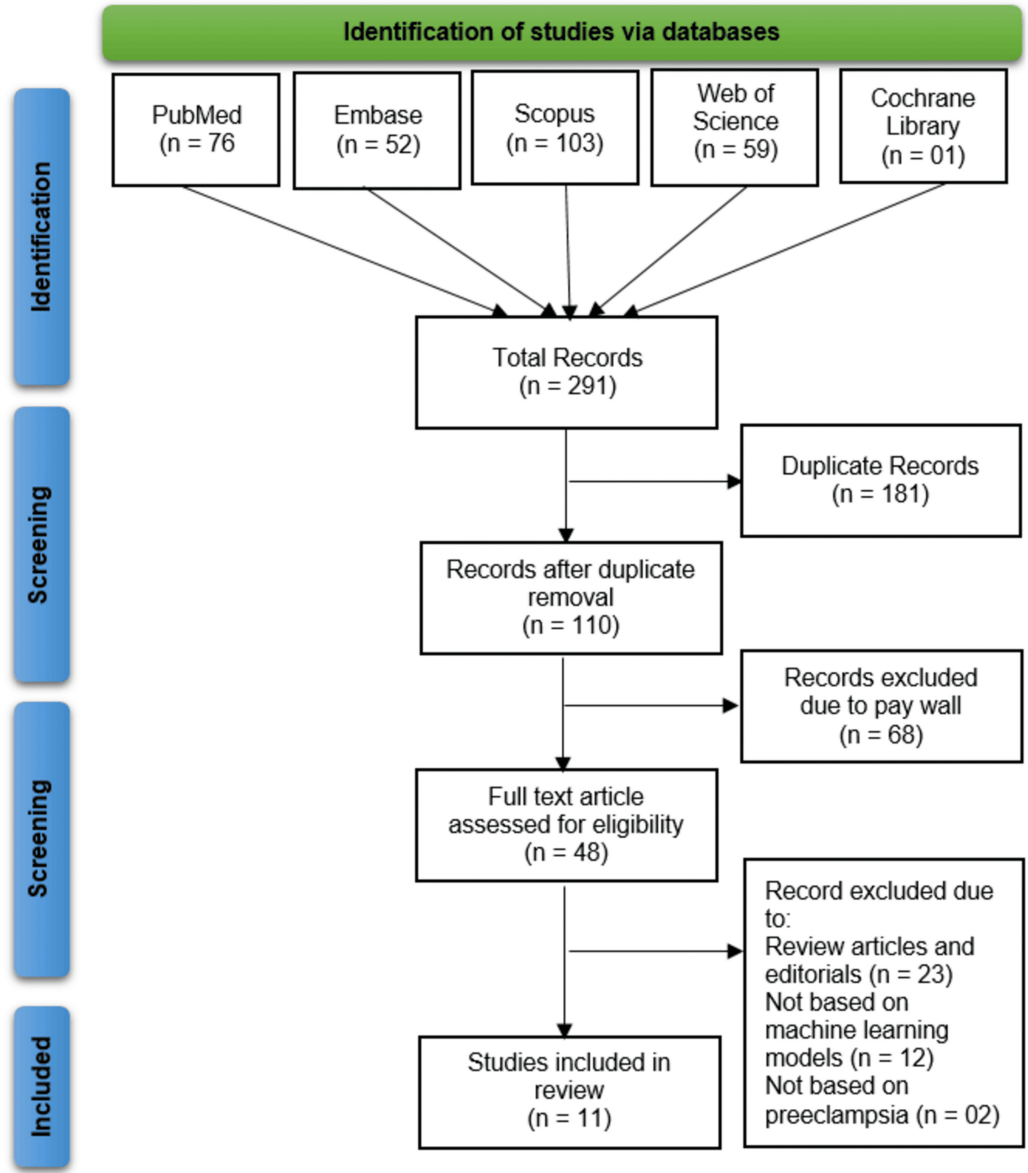
Study selection flowchart showing the number of records identified, screened, assessed for eligibility, and included in the systematic review

Following PRISMA 2021 guidelines, our systematic search across five databases (Cochrane Library (n = 1), Web of Science (n = 59), Scopus (n = 103), PubMed (n = 76), and Embase (n = 52)) initially identified 291 records. After removing 181 duplicates and excluding 68 pay-walled articles during title/abstract screening, we assessed 42 full-text articles, ultimately excluding 23 reviews/editorials, 12 non-ML studies, and two nonpreeclampsia studies, yielding 11 studies for final inclusion. Two independent reviewers conducted this process, resolving discrepancies through consensus to ensure methodological rigor (Figure 1).

Overview of Included Studies

This systematic review analyzed 11 studies that developed and validated ML models for preeclampsia prediction, published between 2020 and 2025. The studies spanned diverse geographic regions, including the USA, South Korea, China, Romania, Mexico, Australia, and Spain, reflecting global efforts to leverage AI in obstetric care. Sample sizes varied significantly, from 233 participants in a Romanian case-control study to 48,250 records in an Australian retrospective cohort. The majority (7/11) employed retrospective designs, while four utilized prospective cohorts or validation studies. Data sources included electronic health records (EHRs), hospital databases, and prenatal screening programs, with input features encompassing maternal characteristics (e.g., age, BMI), medical history, routine lab results (e.g., placental growth factor (PlGF), pregnancy-associated plasma protein A (PAPP-A)), and ultrasound markers (e.g., uterine artery pulsatility index (PI)) (Table 1).

**Table 1:** Overview of studies included in this systematic review AUC: area under the curve; BP: blood pressure; BUN: blood urea nitrogen; CI: confidence interval; DNN: deep neural network; DR: detection rate; DT: Decision Tree; EMR: electronic medical record; EHR: electronic health record; FPR: false-positive rate; FPG: fasting plasma glucose; LR: logistic regression; MAP: mean arterial pressure; ML: machine learning; MoM: multiple of the median; NB: Naïve Bayes; PE: preeclampsia; PI: pulsatility index; PAPP-A: pregnancy-associated plasma protein A; PLGF: placental growth factor; RF: Random Forest; SGB: Stochastic Gradient Boosting; SPR: screen positive rate; SVM: support vector machine; UtA-PI: uterine artery pulsatility index; WBC: white blood cell; XGBoost: Extreme Gradient Boosting; auROC: area under receiver operating characteristic; BMI: body mass index

Study (author, year)	Country	Study design	Sample size	Data source	Input features	AI/ML model used	Outcome predicted	Performance metrics	Validation method
Marić et al., 2020 [8]	USA	Retrospective cohort	16,370 births	Lucile Packard Children’s Hospital, Stanford (2014-2018)	Maternal characteristics, medical history, routine prenatal labs, medication intake (67 variables)	Elastic Net, Gradient Boosting	Preeclampsia, early-onset PE (<34 weeks)	AUC: 0.79 (PE), 0.89 (early PE); Sensitivity: 45.2% (PE), 72.3% (early PE); FPR: 8.1% (PE), 8.8% (early PE)	Cross-validation
Jhee et al., 2019 [11]	South Korea	Retrospective cohort	11,006	Hospital EMR (Yonsei University Hospital)	Systolic BP, BUN, creatinine, platelet count, serum potassium, WBC count, serum calcium, urinary protein	Logistic regression, Decision Tree, Naïve Bayes, SVM, Random Forest, Stochastic Gradient Boosting (SGB)	Late-onset preeclampsia (≥34 weeks)	C-statistics: SGB (0.924), RF (0.894), LR (0.806), NB (0.776), DT (0.857), SVM (0.573); Accuracy (SGB: 0.973); FPR (SGB: 0.009)	Not specified
Li et al., 2021 [12]	China	Retrospective	3759	Electronic health records (EHR) from Xinhua Hospital, Chongming Branch	38 routine clinical parameters (e.g., FPG, mean BP, BMI)	Logistic regression, Random Forest, SVM, XGBoost	Risk of preeclampsia	Accuracy = 0.920, Precision = 0.447, Recall = 0.789, F1-score = 0.571, auROC = 0.955, Brier score reported	Not specified
Liu et al., 2022 [13]	China	Retrospective medical record review	11,472	Prenatal screening records (clinical and lab data)	18 variables: maternal characteristics, medical history, lab and ultrasound results	DNN, LR, SVM, DT, RF (best: RF)	Preeclampsia	RF AUROC: 0.86 (95% CI 0.80–0.92); accuracy: 0.74; precision: 0.82; recall: 0.42; Brier score: 0.17	Cross-validation
Melinte-Popescu et al., 2023 [14]	Romania	Prospective case-control	233	Tertiary Maternity Hospital (Nov 2019-Sep 2022)	Clinical and paraclinical characteristics (1st trimester)	Decision Tree, Naïve Bayes, SVM, Random Forest	Preeclampsia (all types), early-onset PE, moderate and severe PE	Accuracy: DT 94.1%, SVM 91.2%, NB 98.6%, RF 92.8%; moderate/severe: 70.6%–82.4% accuracy	Not specified
Li et al., 2024 [15]	China	Prospective cohort study	4644 (final)	Longitudinal cohort of pregnant women undergoing PE and aneuploidy screening	Maternal age, height, prepregnancy weight, medical history, mean arterial pressure, uterine artery PI, PAPP-A, PLGF	Logistic regression, extra trees, Voting Classifier, Gaussian process, Stacking Classifier	Preeclampsia (overall, term, preterm)	AUC: 0.884 (preterm PE), 0.856 (preterm PE, ML models), 0.797 (all PE); DR at 10% FPR = 0.625 (voting classifier)	Fivefold cross-validation (train-test split 8:2)
Torres‐Torres et al., 2024 [16]	Mexico	Prospective cohort	3050	Pregnant women in Mexico City	Maternal characteristics, MoM of MAP, UtA-PI, PlGF	Elastic-net	Preterm PE (pPE), early-onset PE (ePE), any PE (all-PE)	AUC: 0.897 (pPE), 0.963 (ePE), 0.778 (all-PE); DR at 10% FPR: 76.5%, 88.2%, 50.1%	Training, validation, and test set split
Tiruneh et al., 2024 [17]	Australia	Retrospective cohort	48,250	Large health service network (2016-2021)	Maternal clinical and medical characteristics: nulliparous status, prepregnancy BMI, prior PE, maternal age, family history of hypertension, comorbidities	Random Forest, XGBoost, logistic regression	Preeclampsia (PE)	RF: AUC 0.84 (95% CI 0.82-0.86), Accuracy 0.79; XGBoost: AUC 0.77 (95% CI 0.76–0.79); LR: AUC 0.75 (95% CI 0.74–0.76), perfect calibration	70/30 train-test split
Gil et al., 2024 [18]	Spain (model developed in the UK)	Validation study (prospective cohort)	10,110	PREVAL study (first-trimester PE screening)	Maternal risk factors, MAP, UtA-PI, PlGF, PAPP-A	Fully connected neural network	Early PE (<34 wks), Preterm PE (<37 wks), All PE	AUCs: 0.920 (early), 0.913 (preterm), 0.846 (all); DRs at 10% SPR: 84.4% (early), 77.8% (preterm), 55.7% (all)	External validation using the Spanish dataset
Lv et al., 2025 [19]	China	Retrospective analysis	1040	Clinical data of pregnant women	Prepregnancy BMI, number of pregnancies, mean arterial pressure, smoking, alpha-fetoprotein, and methods of conception	Logistic regression, XGBoost, Random Forest, SVM, artificial neural network	Early-onset preeclampsia	XGBoost: AUC = 0.963 (train), 0.936 (test); F1 = 0.554 ± 0.068 (train), 0.488 ± 0.082 (test)	Resampling method (internal validation)
Tiruneh et al., 2025 [20]	Australia (South-East Melbourne)	Temporal validation of existing models	12,549 singleton pregnancies	Routinely collected antenatal data from three maternity hospitals (Jul 2021-Dec 2022)	Not explicitly stated	XGBoost (Model 1), Random Forest (Model 2), logistic regression (Model 3)	Preeclampsia prediction	AUC: Model 1 = 0.75, Model 2 = 0.71, Model 3 = 0.76; calibration slopes: 1.15, 0.62, 1.02	Temporal validation

ML Models and Performance Metrics

The studies evaluated a wide array of ML algorithms, with ensemble methods (e.g., Random Forest, XGBoost) and logistic regression being the most common. Maric et al. [8] reported an AUC of 0.89 for early-onset preeclampsia prediction using Elastic Net and Gradient Boosting, while Jhee et al. [11] achieved superior performance (AUC: 0.973) with Stochastic Gradient Boosting for late-onset cases. Notably, Li et al. [12] and Liu et al. [13] demonstrated the robustness of extreme gradient boosting (XGBoost) and Random Forest, respectively, with AUCs of 0.955 and 0.86. Performance metrics extended beyond AUC, including sensitivity (45.2-72.3%), specificity, F1 scores (0.42-0.79), and detection rates at 10% false-positive rates (FPRs: 50.1-88.2%). For instance, Gil et al. [18] validated a neural network model externally, achieving an AUC of 0.920 for early-onset preeclampsia with an 84.4% detection rate.

Key Predictors and Clinical Applicability

Common predictive features across studies included maternal demographics (age, BMI), medical history (prior preeclampsia, chronic hypertension), and biomarkers (mean arterial pressure, PlGF, PAPP-A). Torres-Torres et al. [16] highlighted the utility of first-trimester multimodal data (MAP, UtA-PI, PlGF MoM) in achieving an AUC of 0.963 for early-onset cases. Temporal validation studies, such as Tiruneh et al. [17], underscored the importance of model generalizability, with AUCs ranging from 0.71 to 0.76 across different ML approaches. However, heterogeneity in predictor selection and outcome definitions (e.g., early vs. late-onset preeclampsia) limited direct comparisons.

Risk of Bias

The PROBAST evaluation demonstrated that seven studies [8,13,15-19] exhibited low overall risk of bias. These studies maintained rigorous standards across all domains, particularly in outcome definition and participant selection. In contrast, three studies were rated as high risk due to analytical limitations: Jhee et al. [11] and Li et al. [12] showed potential overfitting issues, while Melinte-Popescu et al. [14] had insufficient handling of missing data. Tiruneh et al. [20] received a moderate risk rating due to unclear predictor selection methods. Notably, all studies, regardless of overall risk, demonstrated low bias in outcome assessment, reflecting consistent and objective diagnostic criteria for preeclampsia. These results underscore the importance of robust analytical methodologies in future research to enhance the clinical utility of machine learning prediction models (Table 2).

**Table 2:** Risk-of-bias assessment results using PROBAST tool PROBAST: Prediction Model Risk-of-Bias Assessment Tool

Study	Participants	Predictors	Outcome	Analysis	Overall risk of bias
Marić et al., 2020 [8]	Low	Low	Low	Low	Low
Jhee et al., 2019 [11]	Low	Low	Low	High	High
Li et al., 2021 [12]	Low	Low	Low	High	High
Liu et al., 2022 [13]	Low	Low	Low	Low	Low
Melinte-Popescu et al., 2023 [14]	Low	Low	Low	High	High
Li et al., 2024 [15]	Low	Low	Low	Low	Low
Torres‐Torres et al., 2024 [16]	Low	Low	Low	Low	Low
Tiruneh et al., 2024 [17]	Low	Low	Low	Low	Low
Gil et al., 2024 [18]	Low	Low	Low	Low	Low
Lv et al., 2025 [19]	Low	Low	Low	Low	Low
Tiruneh et al., 2025 [20]	Low	Unclear	Low	Low	Moderate

Comparative Analysis of Model Performance

Tree-based models (Random Forest, XGBoost) consistently outperformed traditional regression and SVM in AUC and accuracy. For example, Lv et al. [19] reported an XGBoost AUC of 0.963 for early-onset prediction, compared to 0.75-0.77 for logistic regression in other studies. However, simpler models like Elastic Net [8] demonstrated competitive performance with fewer variables, suggesting a trade-off between complexity and clinical utility (Table 3).

**Table 3:** Comparative performance metrics of machine learning models for preeclampsia prediction across included studies SVM: support vector machine; AUC: area under the curve; FPR: false-positive rate; RF: Random Forest; PE: preeclampsia; LR: logistic regression

Study (author, year)	Best performing model	AUC (95% CI or range)	Key metrics	Comparison to other models
Marić et al., 2020 [8]	Elastic Net	0.89 (0.84-0.95)	Sensitivity: 72.3%, FPR: 8.8%	Outperformed Gradient Boosting for early-onset PE
Jhee et al.,2019 [11]	Stochastic Gradient Boosting (SGB)	0.973	Accuracy: 97.3%, FPR: 0.9%	Superior to RF (AUC: 0.894) and SVM (AUC: 0.573)
Li et al., 2021 [12]	XGBoost	0.955	Accuracy: 92%, F1 score: 0.571	Beat LR (AUC: ~0.80) and SVM (AUC: ~0.75)
Liu et al., 2022 [13]	Random Forest (RF)	0.86 (0.80-0.92)	Precision: 0.82, recall: 0.42	RF outperformed DNN and SVM (AUC: <0.80)
Melinte-Popescu et al., 2023 [14]	Naïve Bayes	Accuracy: 98.6%	Moderate/severe PE: 70.6-82.4% accuracy	NB surpassed DT (94.1%) and SVM (91.2%)
Li et al., 2024 [15]	Voting Classifier	0.884 (preterm PE)	DR at 10% FPR: 62.5%	Outperformed extra trees (AUC: 0.856)
Torres‐Torres et al., 2024 [16]	Elastic-net	0.963 (early PE)	DR at 10% FPR: 88.2%	Superior to other models for early PE prediction
Tiruneh et al., 2024 [17]	Random Forest	0.84 (0.82-0.86)	Accuracy: 79%, perfect calibration	RF beat XGBoost (AUC: 0.77) and LR (AUC: 0.75)
Gil et al., 2024 [18]	Neural Network	0.920 (early PE)	DR at 10% FPR: 84.4%	External validation showed robust performance
Lv et al., 2025 [19]	XGBoost	0.963 (train)	F1 score: 0.554 ± 0.068	Outperformed RF and SVM in internal validation
Tiruneh et al., 2025 [20]	XGBoost (Model 1)	0.76	Calibration slope: 1.02	Model 1 surpassed RF (AUC: 0.71) and LR (AUC: 0.75)

Discussion

The integration of ML in preeclampsia prediction represents a significant advancement in obstetric care, as demonstrated by the findings of this systematic review. Our analysis of 11 studies reveals that ML models, particularly ensemble methods like XGBoost and Random Forest, achieve consistently high predictive performance, with AUC values ranging from 0.84 to 0.973. These results compare favorably with traditional prediction methods, which typically report AUCs between 0.64 and 0.96 in conventional statistical models, as noted by Al-Rubae et al. [21] in their systematic review of preeclampsia prediction tools. The superior performance of ML approaches can be attributed to their ability to handle complex, non-linear relationships among multiple predictors, a capability particularly relevant for preeclampsia given its multifactorial pathogenesis. Studies such as Marić et al. [8] and Jhee et al. [11] demonstrated that ML models could effectively integrate diverse data types, including maternal characteristics, medical history, and biochemical markers, to achieve prediction accuracies surpassing those of conventional risk scoring systems.

The exceptional performance of ensemble methods in our review aligns with broader trends in medical AI research. XGBoost, in particular, emerged as a consistently top-performing algorithm across multiple studies [12,17,19], likely due to its built-in regularization features and ability to handle imbalanced datasets, a common challenge in preeclampsia prediction where case numbers are typically much smaller than controls. This finding corroborates previous research by Akazawa et al. [22] on ML applications in obstetric outcomes, which similarly found that tree-based ensemble methods outperformed other approaches. However, it's noteworthy that simpler models like Elastic Net [8,16] also demonstrated competitive performance while using fewer variables, suggesting potential advantages in clinical implementation where model interpretability and parsimony are valued. This observation echoes the conclusions of Brunelli and Prefumo [23], who emphasized the importance of balancing model complexity with clinical utility in their quality assessment of first-trimester prediction models.

The timing of prediction emerged as a critical factor in model performance. Studies focusing on early-onset preeclampsia (<34 weeks), such as Maric et al. [8] and Gil et al. [18], generally reported higher AUC values (0.89-0.92) compared to those predicting all-type or late-onset preeclampsia. This may reflect both the greater clinical severity and more distinct biomarker profiles associated with early-onset disease. The incorporation of first-trimester biomarkers like PAPP-A and PlGF, as implemented in Gil et al. [18] neural network model (AUC 0.92), appears particularly promising and aligns with the growing body of evidence supporting early biomarker use [24]. However, the variability in prediction windows across studies (ranging from first trimester to late pregnancy) makes direct comparisons challenging and highlights the need for standardized outcome definitions in future research, a limitation previously identified by O'Gorman et al. [25] in their work on competing risks models.

Our findings regarding predictor variables largely confirm established knowledge about preeclampsia risk factors while revealing some novel insights. Commonly selected features across high-performing models included mean arterial pressure, nulliparity, prior preeclampsia history, and BMI, mirroring the risk factors identified in clinical guidelines [26]. However, several studies also identified less conventional predictors, such as serum potassium levels [11] and alpha-fetoprotein [19], suggesting ML may uncover previously underappreciated associations. This capability to detect complex, non-linear relationships between variables represents a key advantage of ML over traditional statistical approaches, as noted by Sircar et al. [27] in their discussion of preeclampsia pathogenesis. Nevertheless, the heterogeneity in predictor selection across studies, with some models using as few as 18 variables [13] and others incorporating over 60 [8], underscores the need for consensus on core predictor sets to facilitate model comparison and clinical implementation.

The review also reveals important considerations regarding model validation and clinical applicability. While most included studies reported internal validation through methods like cross-validation, only three [17,18,20] conducted external or temporal validation, a critical step for assessing real-world performance. This finding aligns with concerns raised by Al-Rubae et al. [21] about the lack of external validation in existing prediction models. The study by Gil et al. [18] is particularly noteworthy, as it successfully validated a UK-developed neural network model on a Spanish cohort, achieving maintained performance (AUC, 0.92), thus demonstrating cross-population applicability. However, the generally limited attention to model calibration represents a significant gap, as emphasized by Steyerberg et al. [28] in their framework for evaluating prediction models. Tiruneh et al. [17]'s finding of perfect calibration in their large-scale Australian cohort suggests that with adequate sample sizes, well-specified ML models can achieve both discrimination and calibration, both essential for clinical utility.

The risk-of-bias assessment yielded important insights into the methodological quality of current ML research in this field. While most studies demonstrated low risk in participant selection and outcome assessment domains, analytical concerns (particularly around overfitting and missing data handling) were prevalent in several studies rated as high risk [11,12,14]. These findings mirror the methodological deficiencies identified by Brunelli and Prefumo [23] in their evaluation of first-trimester prediction models. The predominance of retrospective designs represents another limitation, as such approaches may not fully capture the dynamic nature of preeclampsia development. Prospective studies like Melinte-Popescu et al. [14], despite their smaller sample sizes, provide valuable insights into real-world implementation challenges that retrospective analyses might miss.

Limitations

Several limitations of this systematic review warrant consideration. First, the heterogeneity in study designs, prediction windows, and outcome definitions precluded meta-analysis and made direct comparisons between models challenging. Second, the exclusion of non-English studies may have introduced language bias, potentially overlooking relevant research from non-English-speaking populations. Third, the rapid evolution of ML techniques means that some included studies may already be surpassed by newer methodologies, a common challenge in AI-related systematic reviews. Fourth, the focus on peer-reviewed publications may have excluded promising preprints or industry-developed models. Finally, the predominance of high-income country data limits the generalizability of findings to low-resource settings where the preeclampsia burden is the highest.

## Conclusions

This systematic review demonstrates that ML approaches show considerable promise for improving preeclampsia prediction, with ensemble methods like XGBoost and Random Forest consistently achieving high performance across diverse populations. The ability of ML models to integrate complex, non-linear relationships between multiple predictors offers advantages over traditional statistical approaches, particularly for early-onset disease prediction. However, significant challenges remain in standardizing predictor sets, improving validation practices, and demonstrating clinical utility in real-world settings. Future research should prioritize prospective, externally validated studies with attention to model calibration and implementation feasibility across diverse healthcare contexts. As the field progresses, collaboration between clinicians, data scientists, and policymakers will be essential to translate these technological advancements into improved maternal health outcomes.
